# The Treatment of Burns

**Published:** 1896-04

**Authors:** 


### The Treatment of Burns.

Bardeleben treats burns after the following plan: The injured part is first thoroughly washed with carbolic acid solution from 2½ to 3 percent, or with a solution of salicylic acid about 3 in 1,000. All the bullæ are then punctured and the serum allowed to escape, after which the whole part is thoroughly dusted with finely-powdered nitrate of bismuth, and a thick layer of cotton wool applied. The latter is changed whenever it is impregnated with the discharges from the wound. If the burn is a very extensive one, the powdered bismuth may be set aside, and a bismuth ointment used instead. The author affirms that with this dressing cicatrization proceeds rapidly, and there is less discomfort than when any other dressing is employed. Despite the large quantities of bismuth that have been applied, no toxic symptoms have been noted in consequence of its use.—*The Medical Age.*

